# Deep mixed model for marginal epistasis detection and population stratification correction in genome-wide association studies

**DOI:** 10.1186/s12859-019-3300-9

**Published:** 2019-12-27

**Authors:** Haohan Wang, Tianwei Yue, Jingkang Yang, Wei Wu, Eric P. Xing

**Affiliations:** 1 Language Technologies Institute, School of Computer Science, Carnegie Mellon University, Pittsburgh, PA USA; 2 Language Technologies Institute, School of Computer Science, Carnegie Mellon University, Pittsburgh, PA USA; 3 Department of Electrical and Computer Engineering, Rice University, Houston, TX USA; 4 Computational Biology Department, School of Computer Science, Carnegie Mellon University, Pittsburgh, PA USA; 5 Machine Learning Department, School of Computer Science, Carnegie Mellon University, Pittsburgh, PA USA

**Keywords:** Marginal epistasis, Mixed model, GWAS, Deep learning

## Abstract

**Background:**

Genome-wide Association Studies (GWAS) have contributed to unraveling associations between genetic variants in the human genome and complex traits for more than a decade. While many works have been invented as follow-ups to detect interactions between SNPs, epistasis are still yet to be modeled and discovered more thoroughly.

**Results:**

In this paper, following the previous study of detecting marginal epistasis signals, and motivated by the universal approximation power of deep learning, we propose a neural network method that can potentially model arbitrary interactions between SNPs in genetic association studies as an extension to the mixed models in correcting confounding factors. Our method, namely Deep Mixed Model, consists of two components: 1) a confounding factor correction component, which is a large-kernel convolution neural network that focuses on calibrating the residual phenotypes by removing factors such as population stratification, and 2) a fixed-effect estimation component, which mainly consists of an Long-short Term Memory (LSTM) model that estimates the association effect size of SNPs with the residual phenotype.

**Conclusions:**

After validating the performance of our method using simulation experiments, we further apply it to Alzheimer’s disease data sets. Our results help gain some explorative understandings of the genetic architecture of Alzheimer’s disease.

## Background

Genome-Wide Association Studies (GWASs) have helped uncover associations between genetic variants and complex traits for more than a decade. The methods for GWA studies first started with the univariate hypothesis testing, and later, many advanced statistical and machine learning methods have been proposed to infer and gain insights into the genetic architectures of the complex traits. For example, linear mixed models are demonstrated with empirical successes in correcting confounding factors raised by population stratification, family relatedness, and cryptic relatedness [1–5], and multivariate regression methods are introduced for modeling the polygenetic effects [6–8]. Integration of these two methods is also introduced to successfully consider polygenicity and confounding factor correction together [9, 10].

Despite promising results have been generated using these approaches, it has been long known that additive effects can explain only part of genetic variations [11]. Epistasis (*i.e.*, interactions between genetic variants) is believed to be a potential source of the unexplained variations [12–15]. Evidence of epistatic interactions has been shown for human complex traits [16–18], suggesting that more potential interactions between genetic variants are to be discovered, which motivates the development of more powerful computational methods.

Epistasis detection is usually highly computational challenging, and thus many efforts have been made by gearing towards developing efficient computational tools for discovering epistasis with different searching strategies, including exhaustive [19–23], probabilistic [24], or prioritized search [25–30]. In addition to these methods that mainly focus on the detection of pairwise interactions of SNPs, a few methods were developed for detecting higher order interactions, and they either rely on probabilistic sampling [31] or ultra-high-performance computing service [32]. Recently, Crawford *et al* proposed an alternative strategy for testing the exact combinations of candidate SNPs. Their method, named MAPIT, tests to identify the SNPs that involved in the epistasis marginally [33]; in other words, their aim to identify the SNPs that are associated with the phenotype in an epistastic manner without revealing the exact combination of these SNPs.

In this paper, continuing with the goal of investigating marginal epistasis, we propose a deep-learning-based method that can implicitly model arbitrary high-order interactions between genetic variants, as well as simultaneously correct confounding effect due to population stratification, family structure, and cryptic relatedness. The central design rationale behind our model is the universal approximation property of deep neural networks [34], which allows neural networks to model arbitrary interactions of the input features (*i.e.*, epistasis). To take advantage of this property, we propose the Deep Mixed Model (DMM). DMM consists of two components: 1) A confounding factor correction component that is a one-dimensional convolutional neural network (CNN) with a large kernel size, thus CNN can focus mostly on the population-wise pattern of data. 2) A variable selection component that mainly consists of a fine-grained Long-short Term Memory (LSTM) model with sparse variable selection methods plugged in; this component is responsible for identifying the SNPs that are associated with the residual phenotype in univariate, polygenetic, or epistastic manners.

We first conduct simulation experiments to demonstrate the superior empirical performance of DMM over competing methods and to inspect and verify the internal working mechanism of DMM. Then we apply DMM to real-world Alzheimer’s disease data sets, and DMM identifies several interesting SNPs. Some of these results are supported through literature surveys, which suggest that our findings, despite explorative at the current stage, may lead to some novel understandings of the Alzheimer’s disease.

## Methods

In this section, we formally introduce our proposed Deep Mixed Model, which is composed of two components, one for confounding factor correction and the other for genetic variants selection. We refer to these two components as *corrector* and *selector* for convenience. We first present the overall concept and then discuss each component in detail.

### Overview

**Fig. 1:**
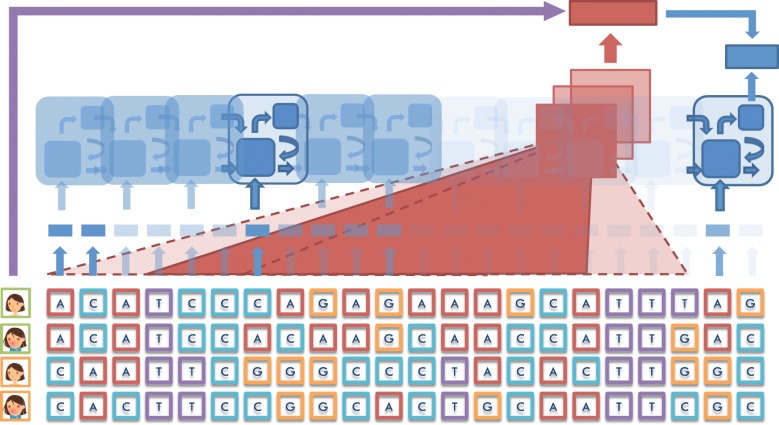
The structure of Deep Mixed Model (DMM), which consists two components: 1) the red component is a convolutional neural network with a large kernel size that scans over the SNP sequence to detect the population-level effect; and 2) the blue component is an LSTM with a vanilla network attached to the input that identifies the genetic variants associated with the phenotype

Figure 1 illustrates the main idea of our proposed Deep Mixed Model, which consists of two components: 1) the red part of the figure represents the *corrector*, which is a convolutional neural network with a large kernel size. The large kernel size forces the CNN to focus more on the overall pattern represented by the genetic variants, instead of variations of specific SNPs, and thus resulting in a population effect estimator; and 2) the blue part of the figure represents the *selector*, which is an LSTM with a sparse vector attached at the input. We will discuss the details of these two components immediately after this overview.

In this paper, we use $\mathbf {X} \in \mathcal {R}^{n \times p}$ to denote the SNP array in our study, $\mathbf {y} \in \mathcal {R}^{n \times 1}$ to denote the phenotype, where *n* represents the number of samples, and *p* represents the number of SNPs. We use **β** to denote effect sizes for fixed effects and **u** to denote effect sizes for random effects. The dimension of **β** and **u** can be inferred from the context. We use *f*(·;**δ**) to denote the *corrector*, and **δ** stands for the corresponding parameters. Similarly, we use *h*(·;**θ**) to denote the *selector*, and **θ** stands for the parameters. *g*^−1^(·) denotes the inverse linkage function of a generalized linear model. *ε* denotes natural noise which is negligible in most cases throughout this paper.

### The confounding factor correction component (the corrector)

To account for confounding factors, we propose a one-dimensional convolutional neural network that estimates the population-level effects and further calculates the residual phenotype after removing these effects. To enforce that CNN primarily focuses on estimating population-level effects, we adopt a large size of the convolutional kernel, based on the understanding that a kernel with large size will encourage the network to learn high-level conceptual representations – rather than detailed variations – of the data [35]. Different from the conventional mixed models that estimate the second-order statistics (variance) raised by confounding factors using the kinship matrix [36], the *corrector* directly operates on the data matrix and estimates the first-order statistics, which is also sufficient in helping remove the confounding factors, justified by the resemblance between a linear mixed model and a ridge regression (Wang H, Aragam B, Xing EP: Statistical analysis of linear mixed model for gwas. in preparation).

### The fixed-effect estimation component (the selector)

For the component that is responsible for selection of genetic variants, we choose the LSTM. Instead of feeding the data directly into the LSTM, we add a one-dimension weighing vector for SNPs; by doing so, the magnitude of the corresponding value of the weighting vector can directly reflect the importance of the genetic variants evaluated by the model, as shown by [37]. More specifically, we can decompose the *selector* as:

$$\begin{array}{*{20}l} h(\mathbf{X}_{i} ; \mathbf{\theta}) = l(\mathbf{X}_{i} \odot \mathbf{\omega}; \mathbf{\iota}) \end{array} $$

for *i*^th^ sample, where ⊙ denotes element-wise product, **ω** denotes the weighting vector, and *l*(·;**ι**) denotes the generic LSTM module whose parameters are denoted as **ι**. The fixed-effect estimation component consists of both **ω** and *l*(·;**ι**), and we denote the parameters as **θ**=[**ω**;**ι**].

### Algorithm

The algorithm for solving DMM splits into two steps: 1) estimating the parameter **δ** for the *corrector* (*f*(·;**δ**)), and 2) estimating the parameter **θ** for the *selector* (*h*(·;**θ**)). The estimation of **δ** can be done straightforwardly by solving:

1 $$\begin{array}{*{20}l} \hat{\mathbf{\delta}} = \underset{\mathbf{\delta}}{\arg\ \min} c(\mathbf{y}, f(\mathbf{X};\mathbf{\delta}))  \end{array} $$

where *c*(·,·) is a generic cost function; for example, we can use the mean squared loss for data with continuous phenotypes and use the cross entropy loss for case-control data.

With $\hat {\delta }$, we can further estimate *θ* by solving:

2 $$\begin{array}{*{20}l} \hat{\mathbf{\theta}} = \underset{\mathbf{\theta}}{\arg\ \min} c(\mathbf{y}, g^{-1}(h(f(\mathbf{X};\hat{\mathbf{\delta}});\mathbf{\theta})))  \end{array} $$

where *g*(·) can also be chosen based on the understanding of data; for example, a linear function can be used for continuous phenotypic data and a logic function for case-control data.

It is essential to avoid overfitting in genetic studies, especially because the psychiatric genetic data are costly to obtain, and we usually only have a sample size of a couple hundred. To avoid overfitting, we stop the training process before the optimization starts to converge, which is known as early-stopping, a regularization method for neural networks [38, 39]. While both Function 1 and Function 2 are optimized with early-stopping, we empirically notice that, in the simulation experiments, the early-stopping is particularly crucial for optimizing *corrector* since it effectively prevents the CNN from estimating additional (unnecessary) information other than true confounding effects from population-level factors. We notice that the *corrector* only needs to be tuned for about 10 epoches.

The detailed configurations of our method mentioned above are summarized in Table 1. With such configuration, in practice, it takes our method less than an hour to converge on the real data experiment (details to be followed in the “Results” section) with a modern GPU. Our method scales well with the number of samples, but limited with the number of SNPs considered due to the limitation of the memory of GPU or CPU.

**Table 1:** Detailed configurations of the method

Collector (1D-CNN)	Convolutional layer	Num. of Kernels: 16	Kernel Size: 1000 x 1	Padding: Same
		Initializer: Truncated normal initializer		Activation: ReLU
	Pooling layer	Size: 2000	Stride: 2000	
	1st fully-connected layer	Output: 32	Dropout rate: 0.9	
	2nd Fully-connected layer	Output: 1		
Selector (LSTM)	Weighting layer	Num. of units: p (one-to-one layer)		
	Hidden layer	Num. of units: 0.15p		
Optimizer (ADAM)	Learning rate: 0.001	Batch size: 128		
Other hyperparams	Collector’s epoch: 20	Selector’s epoch: 1500		

The architecture and hyperparameters are selected through the experiments with simulated data, and are used without changes for real data experiments

## Results

In this section, we will introduce our experiment results, including the simulation results where we compare our method with competing methods and the findings when we apply the DMM to real data. The TensorFlow experiment scripts to replicate the results are submitted as the Supplement. We also released our script as a tool for the community to apply on other data sets at: https://github.com/HaohanWang/DMM.

### Simulations

#### Competing methods

To evaluate the performance of DMM, we compare it with several existing methods listed as follow:

- UT: The standard univariate testing (Wald testing) with the Benjamini-Hochberg (BH) procedure [40]. This is the most popular approach for testing associations in GWAS, without concerning epistasis or accounting for population stratification.
- LMM: A standard linear mixed model with the BH procedure. This is the most popular approach in GWAS for handling population stratification, but not concerning epistasis.
- Lasso: The *ℓ*_1_-regularized linear regression [41].
- Adaptive Lasso (AL): An extension of Lasso that weighs the regularization term accordingly [7] (enabled by the method introduced in [42] for high-dimensional data).
- Precision Lasso (PL): A novel variant of Lasso that can handle correlated and linearly dependent features commonly used in genomics study [8].
- MAPIT: The marginal epistasis test, a method recently proposed for detecting epistasis in GWAS [33]. We re-implement the method in Python for fair comparison. We also add the BH procedure [40] for false discovery control.
- LSTM: The *selector* in the Deep Mixed Model. We test the performance of this component of DMM without the confounding factor correction component.
- DMM: The method we proposed in this paper. ROC curve is calculated with different thresholds of absolute effect sizes.

#### Data generation

We use SimPop [43] to simulate the SNP array. We simulate *p*=10000 SNPs for *n*=500 or 1000 samples from five different populations with migration behaviors. Each population also unevenly splits into five sub-populations. Therefore, it can be seen as these samples are from 25 regions (denoted as **G**) out of five continents. As we mentioned previously, the SNP array is denoted as **X**. We choose the number of samples to be small to reflect the situation of our real psychiatric data.

We select *k* SNPs to be associated with the phenotype, and to simulate the arbitrary interaction patterns of these SNPs, we set a group size of *t* to group these *k* SNPs into *m* groups (the number of groups *m*=*k*/*t*, where *k* is divisible by *t*), and sample *m* effect sizes: each of them is sample as **β**∼*N*(0,25) (This value of variance is chosen following the suggestion of [44] as an intermediate effect size).

As we mentioned previously in the Introduction, there are plenty of methods that can identify the SNPs that are associated to the phenotype with lower order of interaction manner. Therefore, in the experiment, we focus on experimenting with the remaining situation when the multiple SNPs interact (*t*=5), which is more challenging than usual epistasis experiment set-up. However, our set-up is not contradictive to the real-world setting, as this remaining situation will be met when we regress out the lower-order SNP effects.

To introduce confounders such as population stratification and family structure, we use the regions **G** to affect the phenotypes differently (the effects of these regions are denoted as **γ**, sampled from a Gaussian distribution $N(0, \sigma _{u}^{2})$). The variation of $\sigma _{u}^{2}$ results in a signal-to-noise ratio of 0.25 or 1.0 for **β** in our simulation experiment.

Finally, we have the responses as:

$$\begin{array}{*{20}l} \mathbf{r} = \sum_{i=1}^{m}\left(\prod_{j \in i}\mathbf{X}_{j}\right)\mathbf{\beta}_{i} + \mathbf{G}\mathbf{\gamma} \end{array} $$

where we use the product sign ($\prod $) to denote the interaction of the SNPs. We use the element-wise minimum to simulate the interaction. *j*∈*i* denotes that the SNP (indexed by *j*) out of the *k* associated SNPs that belong to the group *m*. We test the methods with the continuous phenotypes generated as

$$\begin{array}{*{20}l} \mathbf{y}_{c} = \mathbf{r} + \epsilon, \end{array} $$

where *ε*∼*N*(0,1). Additionally, we also transform these continuous responses *r* into binary phenotypes via Bernoulli sampling with the outcome of the inverse logit function (*g*^−1^(·)) over current responses. Therefore, we have:

$$\begin{array}{*{20}l} \mathbf{y}_{b} = \text{Ber}(g^{-1}(\mathbf{r})) \end{array} $$

**Fig. 2:**
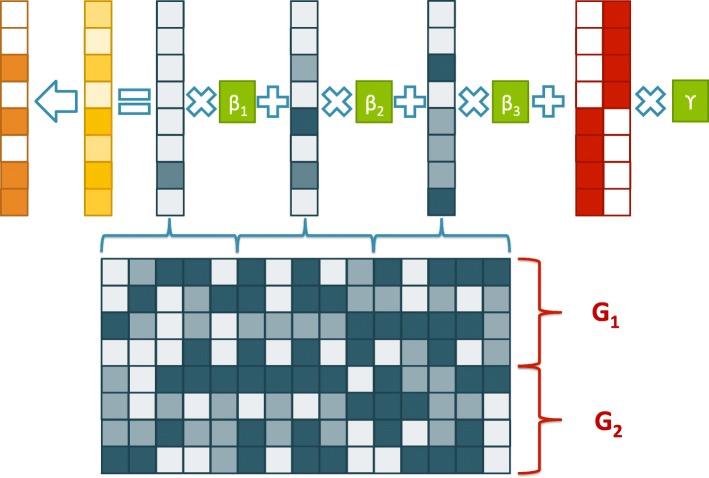
Illustration of the main steps of the simulation data generation process. The dark squares represent the SNP array, with two populations (marked with red descriptions). We group every five SNPs and simulate their interaction, result in one epistatic variable. For each epistatic variable, we introduce an effect size. Summing over the effects introduced by these epistatic variable, together with the effects introduced by population structure, we result in an continuous variable, which will further be transformed into binary phenotype

We experiment on both continuous data **y**_*c*_ and binary data **y**_*b*_. The main steps of this simulation data generation process are conveniently illustrated by Figure 2. Due to the introduction of epistasis, our simulation data becomes extremely difficult for conventional methods to recover the signals, as we will show in the next section.

#### Main simulation results

We test the methods with different settings of different number of samples *n*∈{500,1000} of the effects from confounders $\sigma _{u}^{2} \in \{5, 10 \}$, the number of associated SNPs *k*∈{10,50}, and for continuous phenotype *y*_*c*_ and binary phenotype *y*_*b*_ respectively. There all together 16 different experimental settings, and we run 20 different seeds of each setting. In all these experiments, we investigate the results for the SNPs that are ranked in the first 1000 associated SNPs. Because of the difficulty of our simulation set-up, almost no methods can report meaningful results within top 100 or less reported SNPs.

We evaluate these methods with ROC curves. For testing-based methods (UT, LMM, MAPIT), the ROC curve is plotted by variation of the threshold of p-values. For multivariate regularized methods (Lasso, AL, PL), the ROC curve is plotted with hyperparameters (regularization weight) varying evenly in the logspace from 10^−5^ to 10^5^. For deep learning methods, the ROC curve is plotted with different thresholding of absolute value of estimated *selector* parameter **ω**.

**Fig. 3:**
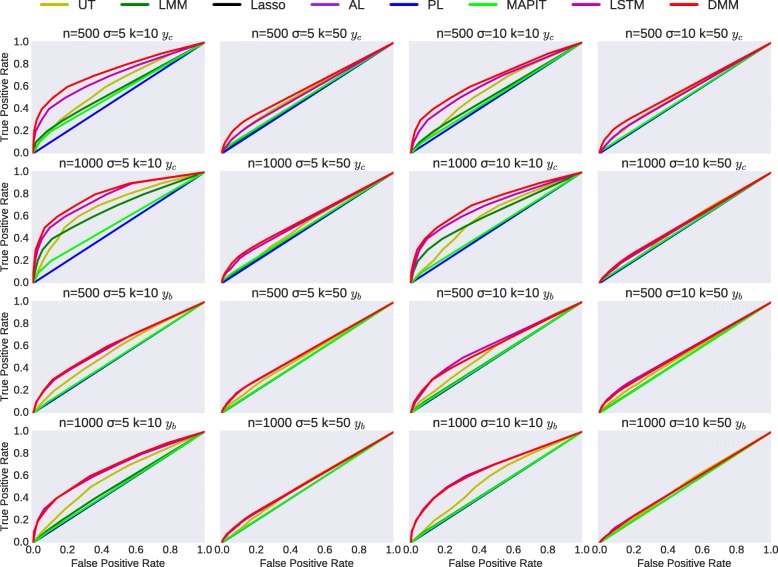
ROC curves of methods in comparison in simulation experiments. The experiment settings vary in different effects introduced from confounders $\sigma _{u}^{2}$ (*e.g.* Confounder Weight, CFW), different number of associated SNPs, and whether the phenotype is continuous *y*_*c*_ or binary *y*_*b*_

Figure 3 shows the simulation results. As we can see, our proposed DMM method has a clear advantage over the competing methods. We can see that almost all the regularized multivariate regression method (Lasso, AL, PL) behave unsatisfyingly in these simulations. We believe this is because of the effects introduced from the confounders. Interestingly, vanilla Wald test generally behave better than other methods despite that it considers neither epistatic effects (not even multivariate effect) nor confounding factors.

By comparing the results in continuous case and the corresponding results in binary case, all these methods behave better in continuous case than in binary case. This is expected because continuous response contains more information. By comparing different settings, the experimental results of methods behave as expected: with less confounding effects, and more samples, the experimental results tend to be better. Also, interestingly, we notice that these methods tend to behave better when there are less associated SNPs to be tested.

To have a more detailed comparison, we also study the averaged Area under ROC of different settings of the experiments corresponding to the results Fig. 3 shows, details shown in Table 2. Notice that all these methods only select top 10% (1000 SNPs) as candidate SNPs for plotting ROC and calculating AUC, which is the primary reason that the regularized multivariate regression method shows a result of exactly 0.5.

**Table 2:** Average AUC value for different methods with different settings on Binary data (B) and Continuous Data (C)

Pheno	*n*	*σ*	*k*	LSTM	DMM	LMM	UT	LASSO	AL	PL	MAPIT
C	500	5	10	0.68	0.73	0.57	0.58	0.50	0.50	0.50	0.56
C	500	5	50	0.54	0.58	0.51	0.55	0.50	0.50	0.50	0.51
C	500	10	10	0.62	0.66	0.54	0.54	0.50	0.50	0.50	0.54
C	500	10	50	0.54	0.58	0.51	0.54	0.50	0.50	0.50	0.51
C	1000	5	10	0.77	0.80	0.68	0.67	0.50	0.50	0.50	0.53
C	1000	5	50	0.56	0.58	0.51	0.52	0.50	0.50	0.50	0.52
C	1000	10	10	0.68	0.71	0.63	0.57	0.50	0.50	0.50	0.51
C	1000	10	50	0.52	0.53	0.51	0.51	0.50	0.50	0.50	0.53
B	500	5	10	0.59	0.60	0.51	0.52	0.50	0.50	0.50	0.51
B	500	5	50	0.55	0.55	0.51	0.52	0.50	0.50	0.50	0.50
B	500	10	10	0.65	0.66	0.52	0.57	0.50	0.50	0.50	0.51
B	500	10	50	0.53	0.54	0.51	0.52	0.50	0.50	0.50	0.50
B	1000	5	10	0.59	0.58	0.51	0.53	0.50	0.50	0.50	0.51
B	1000	5	50	0.55	0.54	0.51	0.52	0.50	0.50	0.50	0.51
B	1000	10	10	0.66	0.65	0.51	0.54	0.50	0.50	0.50	0.51
B	1000	10	50	0.52	0.52	0.50	0.51	0.50	0.50	0.50	0.50

When the phenotype is continuous, DMM shows a clear advantage over other methods, while the LSTM follows in the second place. Therefore, we can safely draw the conclusion that the differences between DMM and the LSTM are due to the ability of the *corrector* for confounding factor correction. Interestingly, there are not many differences between the LMM method and Wald Testing method, which is probably due to the fact that these two methods’ lack of power in identifying the associated signals from arbitrary interaction of the data.

For the binary phenotype case, DMM does not have a clear advantage over just the LSTM, which is related to the known difficulties in the mixed model for correcting the confounding factors in binary data [36].

#### Ability in confounding factor correction

**Fig. 4:**
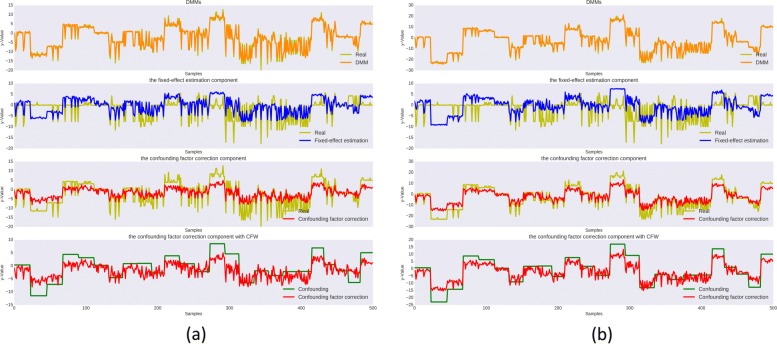
Illustration of internal working pattern of DMM. X-axis shows 500 samples and y-axis shows the phenotype. For each figure, there are 4 sub-figures. The first one shows how the prediction by DMM (orange) fits the true phenotype (yellow). The second shows how the fixed-effect estimation component (blue) fits the phenotype (yellow). The third one shows the how the confounding factor correction component (red) fits the phenotype (yellow), and the fourth one shows how the confounding factor correction component (red) fits the confounding effects (green). (**a**) and (**b**) are two sets of visualizations of the simulation experiments with two different random seeds

In addition to evaluation of end performance of DMM, we continue to investigate the internal working mechanism of DMM. Figure 4 shows how both modules of DMM fit the data. With two examples under different setting of confounding factor weight *σ*, but same setting of *n*=500,*k*=10, and continuous phenotype, we plot the phenotype across 500 samples, and the prediction made by DMM, the *selector*, the *corrector*, and we also plot how the *corrector* fits the confounding factor curve.

As we can see from both figures in Fig. 4, DMM fits the phenotype very well, and we can barely see the differences between these two curves. Further, with the 2^nd^ and 3^rd^ rows, we can see that neither the *selector* nor the *corrector* can predict the phenotype well by itself. At the last row, we can see that the *corrector* tends to capture the pattern of confounding signals, although there are still gaps between what the *corrector* fits and the genuine confounding signals. Also, we can observe that, when confounding signals are stronger, the *corrector* can fit the confounding signals better. These curves verified our design rationale of the DMM: the *corrector* aims to fit the population level confounding signals, while the *selector* fits in the residual signals to pinpoint the associated genetic variants.

### Application to Alzheimer’s Disease

As previous studies indicated the existence of epistasis in Alzheimer’s disease [45], we apply our DMM method to further reveal the genetic architecture of Alzheimer’s disease given the success of our method in simulation data.

We combine two different Alzheimer’s Disease data sets to increase the sample size. The first one is the AD data provided by Alzheimer’s Disease Neuroimaging Initiative (ADNI). We only inspect the individuals that are diagnosed with AD or Normal in their last visit without considering the patients diagnosed with MCI (mild cognitive impairment). There are 477 individuals. The second one is the late-onset AD dataset provided by Harvard Brain Tissue Resource Center and Merck Research Laboratories [46]. The genotype data were generated from 540 patients in an AD cohort matched for age, gender, and post mortem interval, and consists of the measurements for about 500,000 SNPs. The missing values are imputed as the mode of the corresponding SNPs. For both data sets, we only consider the SNPs that reside protein-coding exons according to GENCODE [47]. We further exclude the SNPs on X-chromosome following suggestions of a previous study [48]. There are 6970 SNPs in the experiment.

#### Results

We test the methods on this real data set and apply the models to identify the top 20 SNPs. We report these 20 SNPs in Table 3, where we also list the gene that these SNPs reside in according to GENCODE [47].

**Table 3:** Top 20 SNPs reported by the Deep Mixed Model that are associated with Alzheimer’s disease

Rank	SNP	Chr	Chr. Position	Gene	Rank	SNP	Chr	Chr. Position	Gene
1	rs2360982	14	75764629	*TTLL5*	11	rs7310543	12	69574742	*FRS2*
2	rs4238773	16	53597938	*RPGRIP1L*	12	rs4889798	17	75499805	*TMEM94*
3	rs2424641	20	24665866	*SYNDIG1*	13	rs7959720	12	27333098	*ARNTL2*
4	rs664866	9	137077808	*UAP1L1*	14	rs7036626	9	116425812	*ASTN2*
5	rs6706169	2	165989377	*SCN1A*	15	rs685417	13	32511131	*N4BP2L2*
6	rs7149337	14	50778766	*NIN*	16	rs405281	7	150693280	*GIMAP2*
7	rs12881259	14	90863056	*RPS6KA5*	17	rs10876394	12	51686444	*SCN8A*
8	rs12329001	2	65270990	*ACTR2*	18	rs7639223	3	40260163	*MYRIP*
9	rs13242458	7	99533066	*ZKSCAN5*	19	rs12488539	3	57561864	*PDE12*
10	rs13063312	3	48636988	*CELSR3*	20	rs10402233	19	40472690	*SPTBN4*

Due to the difficulties in verifying epistasis results, we mainly discuss the results reported in Table 3. However, although most other GWA studies that verify their results through comparison to GWAS Catalog [49], our results are not directly comparable there because most findings in GWAS Catalog are conducted through univariate testing methods. Therefore, we do not expect most of our identified SNPs appear in the GWAS Catalog, which creates a challenge in verifying these reported SNPs. As a result, instead of matching these identified SNPs with GWAS Catalog database for verification, we validate these SNPs through the literature search. Because the community is still learning the functionalities of every single SNP, we study the genes these SNPs reside in as a verification of the genuineness of our discoveries. However, one should be aware that although many pieces of evidence will be presented in the following paragraphs, the evidence only directly supports the relationship between the gene these SNPs reside in and the phenotype, and indirectly serves as the verification that our discovered SNPs are authentic. To the best of our knowledge, this literature-search methodology is the best we can do due to the goal of our proposed model.

Several of these genes have been previously reported to be directly related to Alzheimer’s disease. The 5^th^ SNP resides in the gene *SCN1A*. *SCN1A* is reported to affect the neural activity of the aging brain [50]. The 10^th^ SNP resides in the gene *CELSR3*, which is related to brain development, learning and memory behavior processes in aging mice [51]. The 13^th^ SNP lies in the gene *ARNTL2*, which has been reported to be associated with Alzheimer disease in Chinese population [52], although the report focused on another SNP within the gene. The 17^th^ SNP resides in the gene *SCN8A*, which is one of the few genes that have been reported to be associated with Alzheimer’s disease through pathway analysis in mouse model [53]. The 18^th^ SNP resides in gene *MYRIP*, which is also repoted to be related with Alzheimer’s disease [54]. The 20^th^ SNP lies in the gene *SPTBN4*, which is also reported as a target gene from independent study on other data sets in through DNA methylation map [55].

Several other genes that have not been reported to be directly related to Alzheimer’s disease also function in the cognitive activities. For example, the 8^th^ SNP resides in the gene *ACTR2*, which is identified to be associated with language impairment through copy number analysis [56]. The 12^th^ SNP resides in the gene *TEME94*, whose variants are associated with neurodevelopmental delay [57]. The 14^th^ SNP lies in the gene *ASTN2*, which is involved in the neural development [58].

To sum up, these verifications suggest that our identified SNPs and the combinations, although explorative, may reveal some new understandings of Alzheimer’s disease. These results also suggest the effectiveness of DMM in identifying the SNPs that contribute to a phenotype with an arbitrarily high order manner.

## Discussion

We also noticed some limitations of our method, for example, the scalability of our method is limited by the memory the GPU. With a modern GPU, our method can only scale up to around 10k SNPs with our current setting. However, as our method only requires a few epoch on the real-world data, a direct fix will be to run our method on CPU clusters instead.

## Conclusions

Following the recent popularity deep learning gains in genetic applications [59], in this paper, we take advantage of the universal approximation property of neural network to build a method that can model the epistasis with arbitrary order of interaction without explicit identifying the combination of SNPs. We built a fixed-effect estimation component that mainly consists of an LSTM, which is well-known for its ability in extracting signals from sequential data. This component is used to identify the associated genetic variants from data. Further, to help eliminate the signals from confounding factors before fixed-effect estimation, we also introduce a confounding factor correction component (a CNN) that helps to remove the effects raised by factors such as population stratification.

Through simulations, we verify the superior performance of our methods over existing methods with simulated data with high-order interaction of SNPs. We further apply our method to Alzheimer’s disease data sets and report the SNPs our method filters (and combinations identified later by testing methods). Many of these findings, although explorative, are supported by our literature search verification, thus may reveal some new understandings of Alzheimer’s disease.

## Data Availability

The implementation and datasets used and analysed during the study are available from the corresponding author on reasonable request.

## Abbreviations

AD: Alzheimer’s disease
DMM: Deep mixed model
GWAS: Genome wide association studies
LMM: Linear mixed model
MAF: Minor allele frequency
SNP: Single nucleotide polymorphism
